# Chimeric antigen receptor T-cell therapy for relapsed and refractory thyroid cancer

**DOI:** 10.1186/s40164-022-00311-z

**Published:** 2022-09-22

**Authors:** Jing Ding, Deyu Li, Xingchen Liu, Hu Hei, Baoxi Sun, Dongmin Zhou, Keshu Zhou, Yongping Song

**Affiliations:** The affiliated Cancer Hospital of Zhengzhou University, Henan Cancer Hospital, 127 Dongming Road, Zhengzhou, 450008 China

**Keywords:** Thyroid cancer, CAR-T, Relapsed and refractory, TSHR

## Abstract

The prognosis of most thyroid cancer patients is excellent, but for those with advanced or metastatic thyroid cancer, effective treatments are still lacking. Chimeric antigen receptor (CAR) T-cell therapy has gained remarkable achievements in hematologic malignancy but shown limited efficacy in solid tumors. In this report, we showed a relapsed and refractory thyroid cancer patient treated with TSHR + CD19 CAR-T, a combination of two 2nd generation CAR-T molecules targeting both TSHR and CD19. This patient finally achieved partial remission at 3 months and was tolerate well to the regimen. Our study suggested that the CAR-T therapy could be a feasible way in treating relapsed and refractory thyroid cancer.

**Supplementary Information:**

The online version contains supplementary material available at 10.1186/s40164-022-00311-z.

To the editor

In 2020, 586,000 new cases of thyroid cancer occurred globally, ranking in 9th place for cancer incidence [1]. For all the thyroid cancers, more than 95% of the patients have been diagnosed with differentiated thyroid cancer (DTC), which derives from follicular thyroid cells. Generally speaking, DTC tends to be biologically indolent compared to most solid tumors. Unfortunately, those relapsed and refractory (R/R) thyroid cancers, which are unresectable, resistant to radioiodine and generally have a poor response to known systemic therapies [2, 3]. Therefore, there is strong unmet clinical need to broaden treatment options for R/R thyroid cancer.

The canonical chimeric antigen receptor (CAR) structure includes single-chain variable fragment (scFv)—domain responsible for antigen recognition, a hinge and transmembrane domain, and a combined co-stimulatory and activation domain that initiates T cell activation. CAR molecules can reprogram T cell to recognize and eliminate tumor cells expressing specific antigens. Chimeric antigen receptor T (CAR-T) cells therapy has gained a remarkable effect in hematologic malignancy [4–6]. However, the application of CAR-T cells in solid tumors remains much less effective due to the tumor microenvironment that impedes the access of CAR-T cells into the solid tumor, demanding a novel strategy [7, 8]. The thyroid-stimulating hormone receptor (TSHR) is a surface glycoprotein receptor, which is highly and homogeneously expressed on most of thyroid cancer [9, 10]. A recent preclinical study that used a CAR T cell with two co-stimulatory domains and targeting the tumor antigen TSHR had demonstrated the safety and potent efficacy in treating differentiated thyroid cancer [10]. Our preclinical data also showed that TSHR CAR-T cells could effectively kill tumor cells expressing TSHR and released high levels of IL-2 IFN-γ, TNF-α, and Granzyme-B compared to regular T cells (Additional file 1: Fig. S1A). In vivo assessment of TSHR CAR-T cell also demonstrated potent killing efficacy (Additional file 1: Fig. S1B, C). These features suggest the potential of TSHR as CAR-T therapy target for the treatment of thyroid cancer. In this study, one patient with R/R thyroid cancer was treated with TSHR + CD19 CAR-T cells, a combination of two 2nd generation CAR-T molecules targeting TSHR and CD19, and consisting of a CD8 transmembrane domain, a 4-1BB costimulatory domain and a CD3ζ signaling domain to evaluate the safety and efficacy.

The patient diagnosed with poorly differentiated follicular papillary carcinoma in August 2013. Then she underwent a bilobectomy of thyroid. After the surgery, the patient was treated with ^131^iodine from October 2013 to January 2014 at local hospital, the outcome was unknown. In September 2014, she was diagnosed with iodine-refractory thyroid cancer. From September 2015 to January 2016, she received five cycles of etoposide and carboplatin in local hospital. In June 2016, she participated in a clinical trial of a multiple targeted tyrosine kinases inhibitor called Anlotinib (CTR20150735) in our hospital, and was withdrew from this trial due to disease progression in September 2017. In March 2019, the patient went to a local hospital for coughing out tissue. Neoplastic cells were detected from this tissue and TSHR positive, indicating metastatic thyroid cancer. A computed tomography (CT) examination reported multiple metastases in both lungs. After failure of multiline treatment, the patient enrolled in this clinical trial in August (ChiCTR1900022620). IHC demonstrated expression of TSHR in > 90% tumor cells with an intensity of +/++ (Additional file 1: Fig. S2).

After a lymphodepleting chemotherapy with cyclophosphamide (600 mg/m^2^/day) and fludarabine (50 mg/m^2^/day) for 3 days, the patient received one dose of TSHR + CD19 CAR-T cells (9.06 × 10^6^cells/kg) on August 20, 2019 (day 0) (Fig. 1A). After the infusion, the patient had fever, and the temperature peaked at 39.6 ℃ on day2 (Fig. 1B), accompanied with tremor and dyspnea, indicating a grade 2 cytokine release syndrome (CRS) [11]. The CAR-T cells expanded robustly and peaked at 3.42 × 10^9^/L on day 6, and remained to be detectable for at least 3 months (Fig. 1C). The robust expansion of TSHR + CD19 CAR-T in this patient was consistent with the expansion of CAR-T cells as described in hematologic malignancies [12]. Increasing CAR vector copy numbers (VCN) were also observed (Fig. 1C). Infusion of TSHR + CD19 CAR-T cells was followed by a rise in the serum inflammatory cytokines, including interleukin-6 peaking at 3033 pg per milliliter on day 3; and interferon-γ peaking at 347 pg per milliliter on day 3 (Fig. 1D). On day 5, 40 mg of methylprednisolone was administered. At the next day, the patient’s finger pulse oxygen continued to decline, accompanying the language expression disfunction, hallucination and tremor of hands. The patient was treated with dexamethasone 10 mg and mannitol 25 mg, and transferred to ICU for comprehensive supportive care. After stable condition, patient was transferred back to the general ward for further consolidation treatment.

**Fig. 1:**
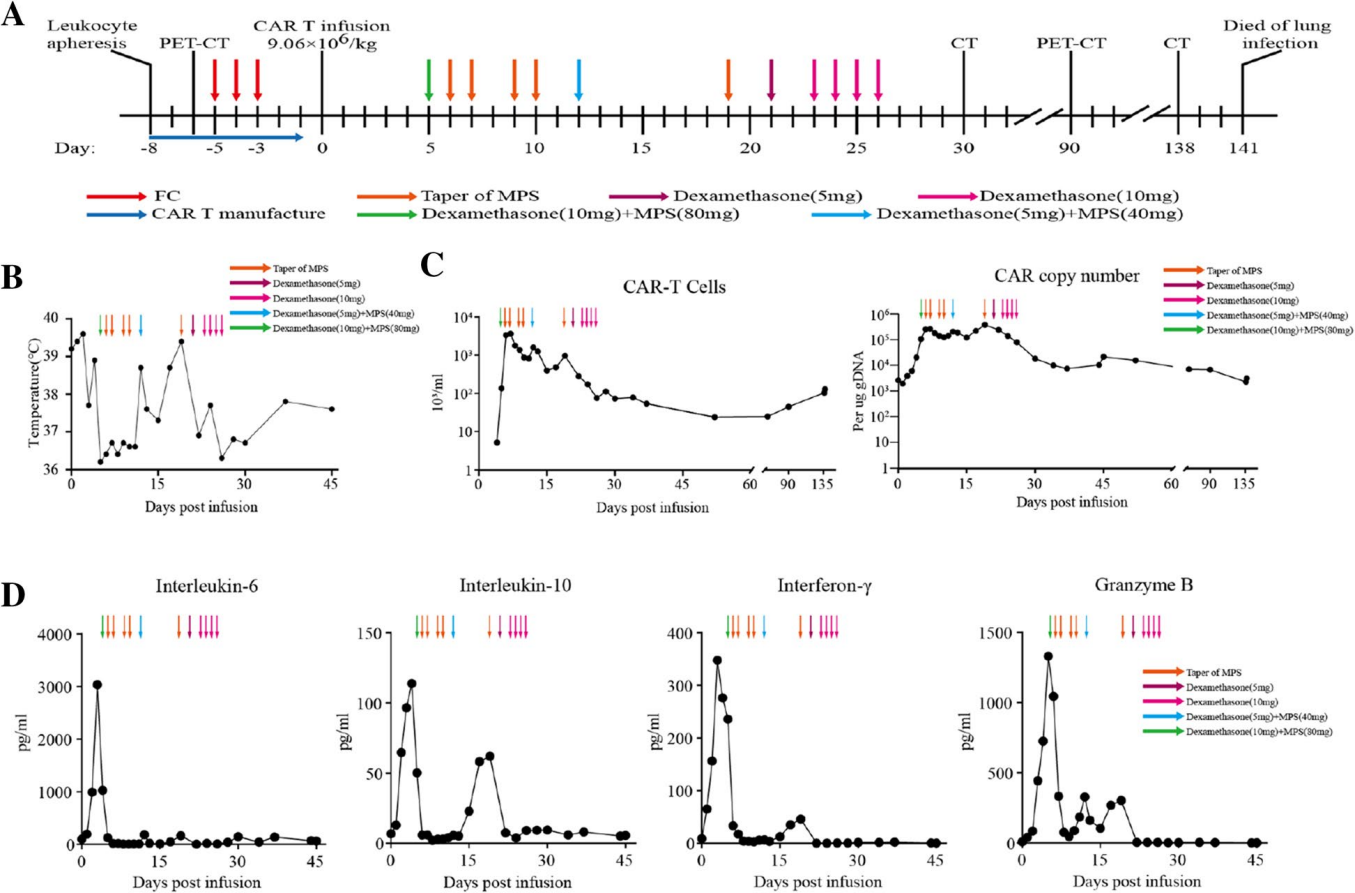
Expansion of Chimeric Antigen Receptor T Cells In Vivo. **A** An overview of the treatment and monitoring of the patient from day − 8 to day 141. CAR-T cells were delivered through intravenous infusion on day 0. The dose of conditioning chemotherapy was fludarabine (30 mg/kg/day) plus cyclophosphamide (300 mg/kg/day). PET-CT, positron-emission tomography-computed tomography. **B** Changes in maximum body temperature per 24-h period. **C** The levels of CAR-T cells quantified by flow cytometry (left) and quantitative real-time polymerase-chain-reaction (center) from day 1 to day 137 after infusion. The y axes of these panels are log10 scales. **D** The dynamic changes of cytokines interleukin-6 and10, interferon-γ, and granzyme B

The whole-body FDG-PET/CT and CT scan were administrated on day 30 post CAR-T infusion, and showed no typical malignant tumor hyper-metabolic lesions. According to the imaging results, the patient acquired stable disease at day 30. Since then, CT scan were administrated every 3 months. The patient acquired a partial remission at day 90 (Fig. 2).

**Fig. 2:**
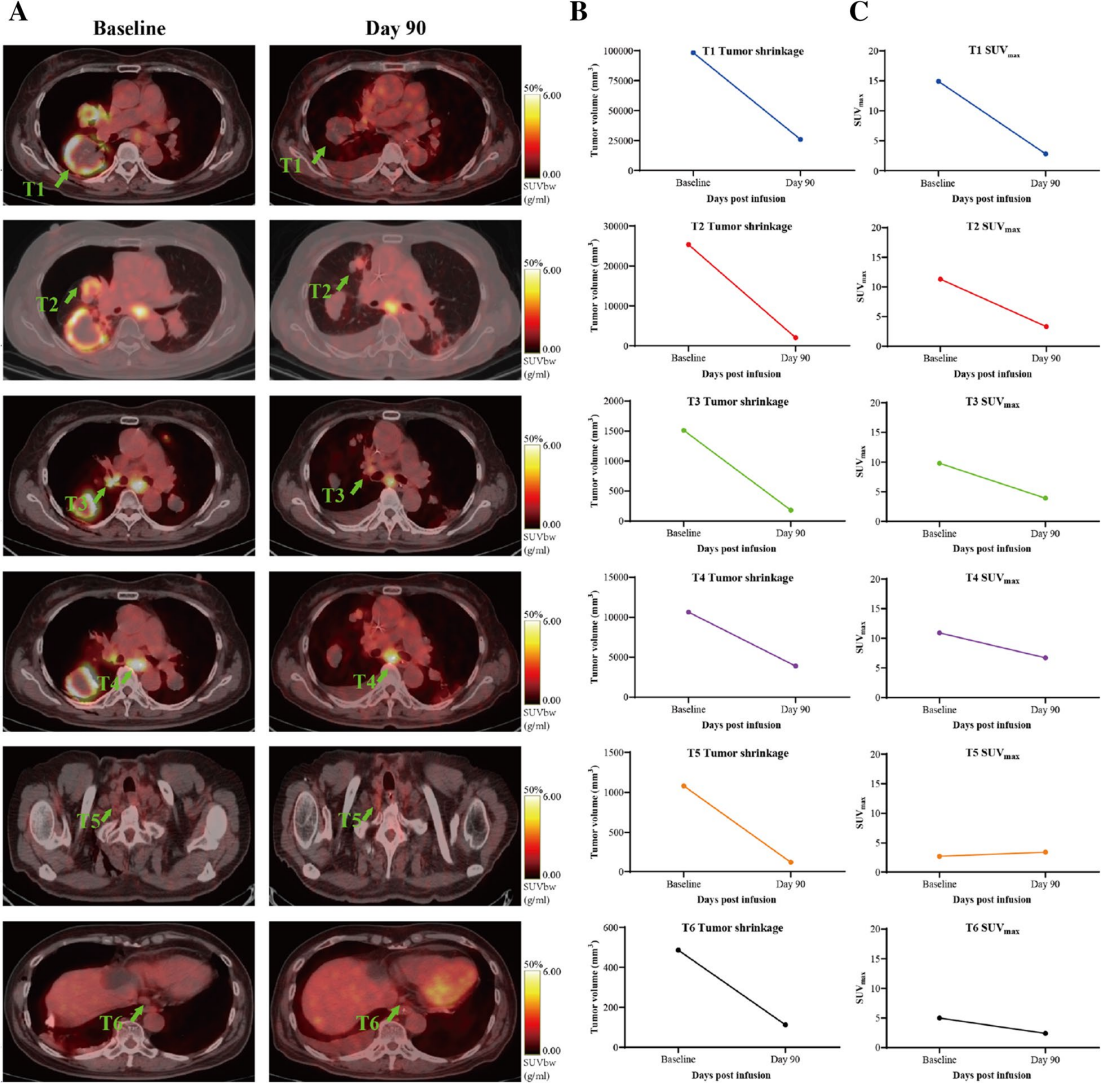
Clinical Response to TSHR19CAR-T Therapy. **A** PET-CT images of the lesions and pathological lymph nodes before (day −8) and after TSHR + CD19 CAR-T infusion (day 90). These illustrated lesions, including a target lesion at the inferior lobe of right lung (T1), a target lesion at the middle lobe of right lung (T2), a non-target pathological lymph node at the hilum of right lung metastasis (T3), a target pathological lymph node at the inferior mediastinal nodes 7 (T4), a non-target pathological lymph node at the tight supraclavicular region (T5), and a non-target lesion at the anterior lower esophagus (T6), were remarkably resolved after TSHR + CD19 CAR-T Cells infusion. All lesions and pathological lymph nodes were shrinking dramatically, achieving a partial remission and a partial metabolic response by day 90 according to RECIST 1.1 and PERCIST 1.0. **B** The changes in tumor volumes (mm3) of the lesions or pathological lymph nodes with CAR-T cell therapy. **C** The changes of the maximum standard unit value (SUV_max_) of the lesions and pathological lymph nodes with CAR-T cell therapy

On January 6, 2020, the patient was diagnosed with “pulmonary infection” in a local hospital, and was transferred to ICU for emergency treatment and died on January 8, 2020. Before the patient died, the efficacy evaluation of thyroid cancer treatment remained PR.

These results indicate that the TSHR + CD19 CAR-T, which induced a robust expansion of anti-tumor CAR-T cells in this patient, could be effective if close monitoring is provided and adverse reactions are treated in time.

## Supplementary Information

**Additional file 1:** Detailed methods and results

## Data Availability

The processed data and analysis codes are available upon reasonable request from the corresponding author.

## Abbreviations

CAR: Chimeric antigen receptor
TSHR: Thyroid stimulating hormone receptor
R/R: Relapsed and refractory
CT: Computed tomography
CRS: Cytokine release syndrome
