# Supplementary material for: Chimeric antigen receptor T-cell therapy for relapsed and refractory thyroid cancer
Source: Exp Hematol Oncol. 2022 Sep 22;11:59. doi: 10.1186/s40164-022-00311-z (PMC9494903; doi:10.1186/s40164-022-00311-z)
Supplement: Supplementary file 1 — Additional file 1: Detailed methods and results [file 40164_2022_311_MOESM1_ESM.docx]

**Chimeric Antigen Receptor T-cell Therapy for relapsed and refractory Thyroid Cancer**

Jing Ding, Deyu Li, Xingchen Liu, Hu Hei, Baoxi Sun, Dongmin Zhou, Keshu Zhou* and Yongping Song*

**Additional file: Detailed methods and results**

**Cell line and cytokine release**

Thyroid cancer cell line 8505C was purchased from the BeNa Culture Collection (BNCC) and were maintained in EMEM (HBSS) medium supplemented with 2mM Glutamine and 1% Non-Essential Amino Acids (NEAA) and 10% Foetal Bovine Serum (FBS). Cells were grown in a humidified incubator at 37 °C in the presence of 5% CO2. TSHR over-expressed cell lines 8505C-TSHR was prepared through infection by recombinant lentiviruses containing the full length of the human TSHR cDNA sequence. Secreted IL-2, IFN-γ, Granzyme B and TNF-α were measured by human cytokine kit (Bio-Rad) according to the manufacturer’s directions. Briefly, we firstly prepared 5-parameter logistic calibration curve of 4 cytokine reference standards from 20pg/ml to 5000pg/ml which were given in the kit. Capture beads of each cytokine were mixed and incubated into each sample and calibration standard with human Th1/Th2 PE signal antibody for 3h. After centrifugation to discard supernatant, samples were re-suspended in wash buffer and were ready for flow cytometry analysis. Different cytokines were gated in flow cytometry. Calibration curve was generated for each cytokine according to the PE signal and the cytokine concentration was read in each sample. Cytokine concentration was normalized to pg/CART cells.

**Animal study**

All the animal studies were conducted in Shanghai Kingbio Biosciences Inc, and the protocols were in compliance with the Institutional Animal Care of Shanghai Kingbio Biosciences Inc. NOG mice were purchased from Shanghai Branch of Beijing Vital River Laboratory Animal Technologies Co. Ltd. The thyroid cancer xenograft model was established by the subcutaneous injection of 5.0x10^6^ 8505C-TSHR cells (D-10). Ten days later (D0), the inoculated mice were randomly divided into the Mock group(n=4), NT group (n = 4) and TSHR CAR-T group (n = 4) and infused with 2.0 x 10^6^ Non-transduced T (NT) cells and 2.0 x 10^6^ TSHR CAR-T cells via their tail vein. The tumor volumes of the mice were measured every day until Day16. Peripheral blood was collected on days 7 and 14 for the analysis of T cell expansion.

**Immunohistochemistry (IHC) staining**

For immunohistochemistry stain, rabbit anti-human TSH receptor antibody (ab218108) was from Abcam and had been qualified for IHC. Tumor biopsies were collected from patients enrolled in the clinical trial. In brief, 4-5 μm sections of tissue samples were dewaxed through a series of alcohol washes and xylene and finally placed into water. Antigen retrieval was performed using a citric acid antigen repair system. The rabbit anti-human TSH receptor antibody was incubated at 1 μg/mL in 4 °C overnight, followed by incubation with a biotin peroxidase detection system (CST, Inc., 8059S). Positive staining was detected using diaminobenzidine.

**Data analysis**

All results were presented with mean +/- SD of technical replicates or mean +/- SEM of biological replicates. Statistical analysis for each experiment was described in the corresponding figure legend. Unpaired two-tailed Student’s t-test was used for comparison of two groups. All pre-clinical and clinical statistical analyses were performed using GraphPad PRISM 9.1.0. ns = not significant, *P < 0.05, **P < 0.01, ***P < 0.001, ****P < 0.0001.

**Results**


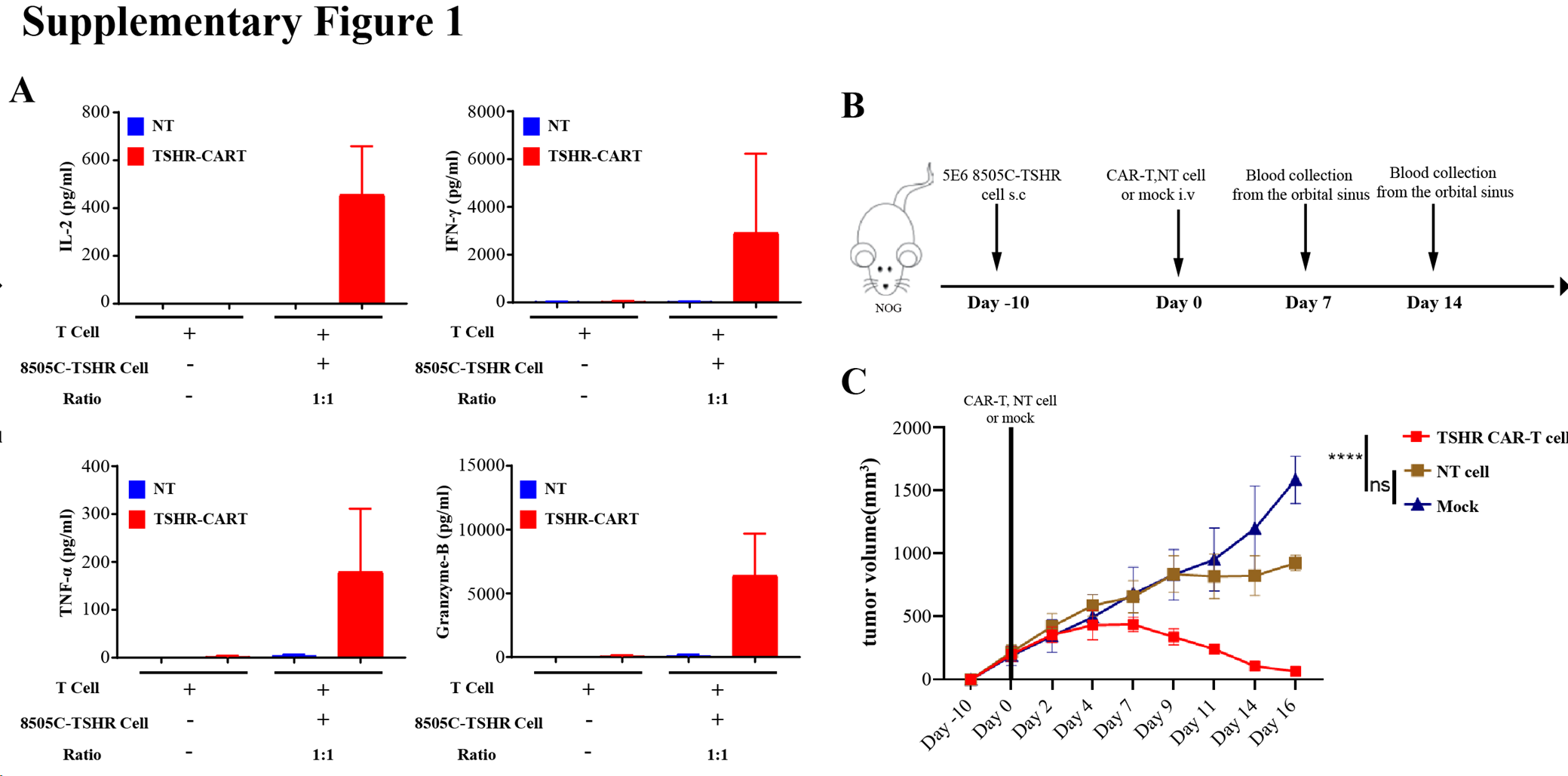


**Fig. S1 TSHR-specific CAR-T cells show strong antitumor activity in vitro and in vivo.** **A**. TSHR CAR-T cells and human non-transduced T (NT) cells were co-cultured for 24 hours with TSHR over-expressed cell line 8505C-TSHR at an E:T ratio of 1:1 followed by determination of levels of IL-2, IFN-γ, TNF-a and Granzyme-B in the supernatants using cytometric bead array (CBA). **B.** Schematic illustration of the in vivo studies. **C.** Tumor volumes in the mice over time. P value < 0.05 = *, < 0.01 = **, < 0.001 = ***, < 0.0001 = ****; P ≥ 0.05 = no significance = ns.


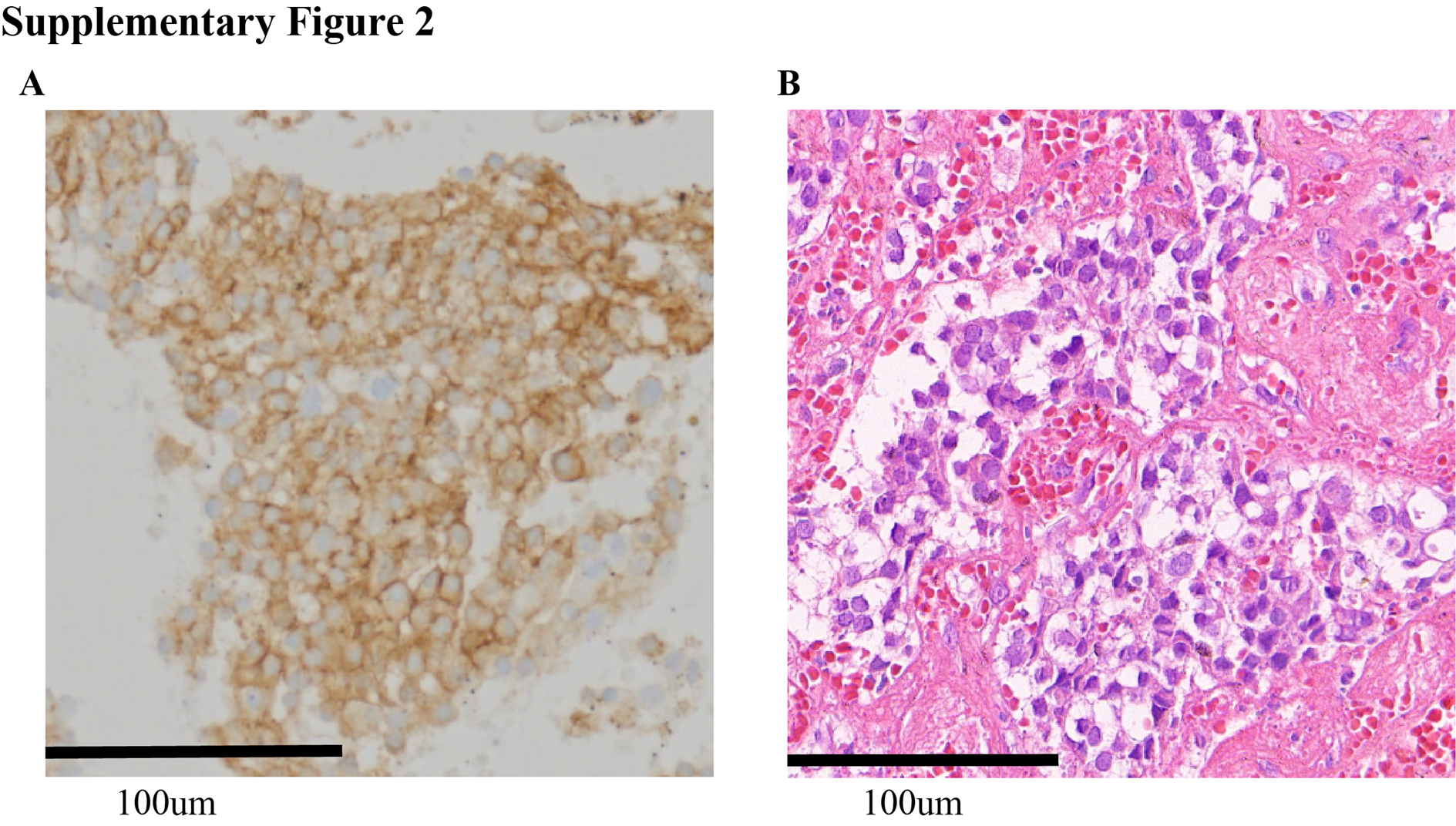


**Figure S2.** **Immunohistochemistry of primary tumor. A**. TSHR immunohistochemistry staining in the patient tumor sample. **B.** Hematoxylin and eosin staining of the patient tumor cells.
